# Cryptococcoma mimicking a brain tumor in an immunocompetent patient: case report of an extremely rare presentation

**DOI:** 10.1590/1516-3180.2017.0046210417

**Published:** 2017-11-06

**Authors:** Aline Lariessy Campos Paiva, Guilherme Brasileiro de Aguiar, Renan Maximilian Lovato, Arthus Vilar Deolindo Zanetti, Alexandros Theodoros Panagopoulos, José Carlos Esteves Veiga

**Affiliations:** I MD. Resident, Discipline of Neurosurgery, Faculdade de Ciências Médicas da Santa Casa de São Paulo (FCMSCSP), São Paulo (SP), Brazil.; II MSc. Attending Neurosurgeon, Discipline of Neurosurgery, Faculdade de Ciências Médicas da Santa Casa de São Paulo (FCMSCSP), São Paulo (SP), Brazil.; III PhD. Attending Neurosurgeon, Discipline of Neurosurgery, Faculdade de Ciências Médicas da Santa Casa de São Paulo (FCMSCSP), São Paulo (SP), Brazil.; IV PhD. Full Professor and Head, Discipline of Neurosurgery, Faculdade de Ciências Médicas da Santa Casa de São Paulo (FCMSCSP), São Paulo (SP), Brazil.

**Keywords:** Cryptococcosis, Brain neoplasms, Meningitis, cryptococcal, Immunocompetence

## Abstract

**CONTEXT:**

Central nervous system (CNS) infectious diseases have high prevalence in developing countries and their proper diagnosis and treatment are very important for public health planning. *Cryptococcus neoformans* is a fungus that may cause several CNS manifestations, especially in immunocompromised patients. Cryptococcal meningitis is the most common type of involvement. Mass-effect lesions are uncommon: they are described as cryptococcomas and their prevalence is even lower among immunocompetent patients. The aim here was to report an extremely rare case of cryptococcoma causing a mass effect and mimicking a brain tumor in an immunocompetent patient. The literature on CNS cryptococcal infections was reviewed with emphasis on cryptococcomas. Clinical, surgical and radiological data on a female patient with this rare presentation of cryptococcoma mimicking a brain tumor are described.

**CASE REPORT:**

A 54-year-old female patient presented to the emergency department with a rapid-onset progressive history of confusion and completely dependency for basic activities. Neuroimaging showed a left occipital lesion and neurosurgical treatment was proposed. From histopathological evaluation, a diagnosis of cryptococcoma was established. She received clinical support with antifungals, but despite optimal clinical treatment, her condition evolved to death.

**CONCLUSIONS:**

Cryptococcal infections have several forms of presentation and, in immunocompetent patients, their manifestation may be even more different. Cryptococcoma is an extremely rare presentation in which proper surgical and clinical treatment should be instituted as quickly as possible, but even so, there is a high mortality rate.

## INTRODUCTION

Cryptococcosis is the most common fungal infection of the central nervous system (CNS) and it occurs mainly among immunocompromised patients.^1^^,^^2^ Transmission occurs especially through inhalation of substances contained in the feces of pigeons and other birds.^1^ It is usually considered to be a differential diagnosis among immunocompromised patients who present difficult-to-treat long-term meningitis.^1^^,^^3^

The lungs are the primary sites of infection. From there, *Cryptococcus* spreads through a hematogenous route and can affect many organs such as the liver and spleen. When this organism passes through the blood-brain barrier, it generally means that the host defenses are compromised. This may be due to human immunodeficiency virus (HIV) infection or to chronic conditions such as renal and vascular diseases.^1^

In rarer cases, *Cryptococcus neoformans* infection may be manifested as neurocryptococcoma, which is a granulomatous CNS lesion that may cause a mass effect.^3^ Few cases have been reported and the differential diagnosis needs to include other neuroinfectious diseases and primary or metastatic tumors.^1^^,^^3^ Dubey et al.^4^ reported only three cases of neurocryptococcoma over a 23-year period, over which 40 granulomatous brain lesions were considered. The treatments included antifungal medications and, in many cases, surgical removal of the lesions.

Here, we describe a rare case of a female patient who did not have any condition that reduced her immunity. The only relevant occurrence in her medical history was the presence of controlled arterial hypertension. She presented to the emergency department with a complaint of confusion that evolved very quickly to completely dependence for daily activities. Brain magnetic resonance imaging (MRI) showed mass-effect lesions in the left occipital lobe. She underwent neurosurgical intervention and meningitis treatment. Initially after surgery, she showed some neurological improvement. However, despite optimal treatment, her condition evolved to death.

## CASE REPORT

A 54-year-old female patient presented to the neurosurgical emergency department, brought by her family, who described a history of rapid and progressive mental confusion (starting around two months earlier, with significant worsening over the last two weeks), leading to complete dependence for basic daily activities such as baths and eating. The only significant condition in her medical history was hypertension. One relevant social factor was that she had grown up on a farm and had had direct contact with several bird species including pigeons.

General and neurological physical examinations revealed that the patient was normotensive but in a poor general condition, was only able to obey simple orders, was restricted to bed (gait was not evaluated because the patient presented general weakness), seemed not to have any motor deficits and did not have any cranial nerves alterations or meningeal signs. It was difficult to perform a complete neurological examination because of her general condition. Based on her history and physical examination, she was categorized as having a score of 50 on the Karnofsky performance scale (KPS).

**Figure 1.**
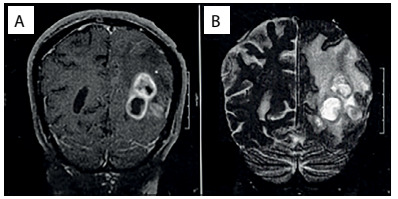
A) Coronal T1-weighted magnetic resonance imaging (MRI) showing enhanced left occipital lesions. B) Coronal T2 MRI showing extensive edema surrounding left occipital lesions.

**Figure 2.**
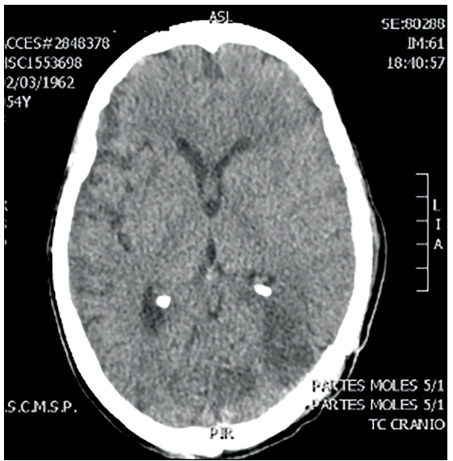
Axial computed tomography performed during the postoperative period showing gross total resection.

**Figure 3.**
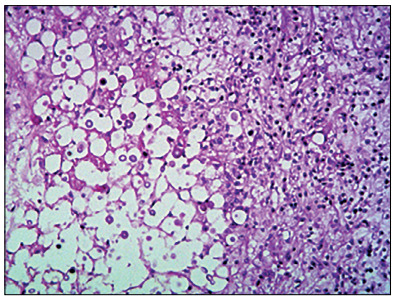
Histopathological analysis on the lesion. On the left side, the deeper part of the lesion shows multiple fungi. On the right side, there are multiple inflammatory cells. Hematoxylin-eosin staining; 40 x.

An MRI scan (Figure 1) performed on the patient a few days before this evaluation was brought with her and this showed two left occipital lesions surrounded by edema. She did not have any other systemic impairment. It was decided to perform surgery to resect these lesions, which had a macroscopic appearance of a solid component with more gelatinous pseudocyst areas inside. A quick check-up was performed, through laboratory tests, including a complete blood count before surgery, chest radiography and an electrocardiogram, and it did not show any abnormalities. Gross total removal was achieved and postoperative computed tomography (CT) scans were performed (Figure 2). In addition, samples from the lesion were sent for histopathological analysis (Figure 3). The result revealed that the lesion consisted of cryptococcoma. Because of this result, a more precise investigation was performed. The patient was found not to have either HIV or hepatitis (both tests were performed twice) or any other immunocompromised conditions. In addition, chest and abdominal CT scans were normal.

After the operation, the patient showed some neurological improvement and, after some days, a lumbar puncture was performed to evaluate the presence of meningitis. The presence of cerebrospinal fluid (CSF) infection was confirmed (large numbers of specimens of *Cryptococcus neoformans* were observed in the samples, along with increased protein levels). Before the puncture, a brain CT scan was performed to rule out any signs of intracranial hypertension or any alteration that might make it impossible to perform this procedure. All punctures revealed increased intracranial pressure (ICP) that progressively worsened (the highest value was 29 cmH_2_O), but she did not present hydrocephalus.

Initially, ventriculoperitoneal shunt was not proposed, but when it was noticed that the ICP levels were progressively increasing and the procedure was indicated, she was seen to present a poor general condition with significant clinical impairment. Proper antifungal treatment consisting of amphotericin B (AmB) and fluconazole was administered, in association with clinical support, because she presented renal failure due to use of many drugs and required hemodialysis. However, despite optimal clinical treatment in the intensive care unit (ICU), comprising antifungal drugs, broad-spectrum antibiotics against pneumonia, electrolyte replacement, hemodialysis and respiratory physiotherapy, the patient’s condition evolved to death due to various complications, including the initial disease, the toxicity of the various medications and bronchoaspiration leading to sepsis.

## DISCUSSION

Cryptococcosis is the most common fungal disease of the central nervous system^1^^,^^2^^,^^3^^,^^4^ and usually affects patients with a condition that reduces their immunological status. It therefore belongs to the group of opportunistic infections. It is most frequently associated with HIV infection^3^ but patients with chronic renal disease, vascular conditions, hepatitis B or C, alcoholism, diabetes mellitus and oncological diseases are also typically more compromised than are immunocompetent patients, who rarely evolve with neurocryptococcosis.

Transmission occurs through inhalation of substances that are present in mammal and bird feces (pigeon feces have been described most frequently). Thus, the lungs constitute the entrance for the disease and pulmonary impairment is usually the first manifestation.^3^ After the fungus enters the body, it can spread to several organs. The liver and spleen are generally affected^3^ and, depending on immunological status, the infection can cross the blood-brain barrier through fungal neurotropism and cause neurological impairment.

Central nervous system cryptococcal infections usually manifest as meningitis, meningoencephalitis, encephalitis or ventriculitis.^5^^,^^6^ These patients usually present with raised intracranial pressure and hydrocephalus, and require shunt procedures. Moreover, specific medications are required to institute the proper treatment. Amphotericin B is the first-line drug and flucytosine or fluconazole are secondary agents.^7^ However, these drugs have severe side effects and, therefore, strict clinical follow-up is required. The main adverse effect is renal failure with electrolyte imbalance.

The World Health Organization (WHO) recommends two weeks of induction treatment with amphotericin B deoxycholate (AmBd) and flucytosine or fluconazole, followed by eight weeks of consolidation treatment with oral fluconazole.^8^ Toxicity analyses on AmB administered at a dose within the currently recommended dose range of 0.7 to 1 mg/kg/day for treatment durations of 5 to 14 days, alone or combined with a second antifungal have been conducted.^7^

Nevertheless, in rare cases, this chronic granulomatous process can lead to formation of a mass (cryptococcoma) that has a tumoral appearance.^9^ Metabolites released by *Cryptococcus* can inhibit the migration and function of leukocytes and promote survival and localized replication of the pathogen, thus facilitating chronic granulomatous inflammation and cryptococcoma formation.^6^ There are a few reports of this condition, mainly in immuno-competent patients who usually did not develop this infection. A systematic review stored in the PubMed database was performed using the MESH terms cryptococcoma and neurocryptococcoma (Table 1). Twelve manuscripts described patients with cryptococcomas, but there were patients both with and without immunosuppressive conditions (Table 2).^4^^,^^9^^,^^10^^,^^11^^,^^12^^,^^13^^,^^14^^,^^15^^,^^16^^,^^17^^,^^18^^,^^19^

**Table 1.** Search of the literature in medical databases for case reports on cryptococcomas. The search was conducted on May 5, 2017

Database	Search strategies	Papers found	Papers related
MEDLINE (Via PubMed)	“Brain Neoplasms”[Mesh] AND “Cryptococcosis”[Mesh] AND Case Reports[ptyp]	28	12

**Table 2.** PubMed-indexed papers in English reporting on cerebral cryptococcomas (MESH terms used: cryptococcoma; neurocryptococcoma)

Author and year	Number of patients	Immuno-suppression	Gender	Adulthood or childhood
Caldemeyer et al.,^12^ 1996	1	No	Female	Child
Ho et al.,^11^ 2005	1	No	Female	Adult
Kanaly et al.,^13^ 2007	1	Yes	Male	Adult
Saigal et al.,^14^ 2006	1	No	Male	Adult
Gologorsky et al.,^15^ 2007	1	No	Male	Child
Sillero-Filho et al.,^9^ 2009	1	Yes	Male	Adult
Patro et al.,^16^ 2009	2	No	1 female and 1 male	Adult
Rai et al.,^18^ 2012	1	Yes	Male	Adult
Jung et al.,^19^ 2012	1	Yes	Male	Adult
Hagan et al.,^10^ 2014	1	No	Female (puerperal)	Adult
Dubey et al.,^4^ 2005	3	Yes	Male	Adult
Hiraga et al.,^17^ 2015	1	No	Female	Adult

Surgical excision and debulking are indicated as adjunct therapies for lesions that are greater than or equal to 3 cm.^11^^,^^20^ The type of neurological manifestation and the findings from neuroimaging depend on the patient’s immunological status. Cryptococcomas are proportionally more likely to occur in immunocompetent patients than in immunosuppressed patients,^11^^,^^21^ but in terms of absolute numbers, this is still an extremely rare condition that is initially difficult to diagnose.^21^

Intraparenchymal cryptococcomas usually present with low signal intensity on T1-weighted MRI and high intensity on T2-weighted images. Solitary cryptococcomas are very rare lesions.^22^ However, despite these radiological hints, this is a very difficult diagnosis to make in non-immunocompromised patients without pulmonary impairment, before histopathological analysis has been conducted. At first, without any precisely known clinical history, these images give rise to other differential diagnoses, such as primary brain neoplasm (especially high-grade gliomas) and secondary lesions due to metastatic tumors. Other CNS infections such as tuberculosis are also diagnoses that need to be considered.

The patient of this paper was extensively investigated but did not have any immunosuppressive condition or pulmonary impairment. The only data that might have helped us to think of the diagnosis of cryptococcoma was the patient’s previous contact with pigeons (which was not mentioned by the family until our team asked about this after the histopathological diagnosis had been established). Because the patient had a neurological dysfunction that developed quickly, which made us think initially that the cause was an aggressive brain tumor, it was decided to perform surgical removal. The histopathological analysis (Figure 3) confirmed all the typical alterations relating to cryptococcosis, and proper treatment was quickly instituted. However, this is a condition with elevated mortality, which also led to death in her case.

## CONCLUSIONS

Diagnosing the tumoral form of cryptococcosis (cryptococcoma) in immunocompetent patients is a challenge even in endemic regions. Primary and secondary brain tumors are usually the first hypotheses in these cases. Proper investigation through anamnesis and imaging can lead to consideration of this diagnosis before the histopathological analysis has been conducted. A neurosurgical approach should be considered in cases of this type of mass lesion, to reduce the compressive factor and thus the intracranial pressure. Despite optimal treatment, this is a condition with high mortality.
